# Developing a Video-Based eHealth Intervention for HIV-Positive Gay, Bisexual, and Other Men Who Have Sex with Men: Study Protocol for a Randomized Controlled Trial

**DOI:** 10.2196/resprot.5554

**Published:** 2016-06-17

**Authors:** Sabina Hirshfield, Martin J Downing Jr, Jeffrey T Parsons, Christian Grov, Rachel J Gordon, Steven T Houang, Roberta Scheinmann, Patrick S Sullivan, Irene S Yoon, Ian Anderson, Mary Ann Chiasson

**Affiliations:** ^1^ Public Health Solutions Research and Evaluation New York, NY United States; ^2^ Center for HIV/AIDS Educational Studies and Training (CHEST) Department of Psychology, and the Graduate Center of the City University of New York (CUNY) Hunter College New York, NY United States; ^3^ Center for HIV/AIDS Educational Studies and Training (CHEST) Graduate School of Public Health and Health Policy City University of New York New York, NY United States; ^4^ Division of Infectious Diseases Departments of Medicine and Epidemiology Columbia University New York, NY United States; ^5^ Rollins School of Public Health Department of Epidemiology Emory University Atlanta, GA United States; ^6^ Smart + Strong New York, NY United States

**Keywords:** eHealth interventions, GBMSM, HIV prevention, HIV disclosure, randomized controlled trial, videos

## Abstract

**Background:**

Gay, bisexual, and other men who have sex with men (GBMSM) accounted for 67% of new US human immunodeficiency virus (HIV) infections in 2012; however, less than 40% of HIV-positive GBMSM are virally suppressed. Preventing transmission from virally unsuppressed men who have condomless anal sex (CAS) with serodiscordant partners is a public health imperative. New HIV infections in GBMSM are attributed in part to online access to sex partners; therefore, low-cost eHealth interventions are a unique opportunity to reach men where they meet partners.

**Objective:**

To describe the protocol of a randomized controlled trial evaluating whether video-based messaging delivered online may lead to reductions in serodiscordant CAS and increased HIV disclosure.

**Methods:**

Sex Positive!^[+]^ is a two-arm, phase III, video-based randomized controlled trial delivered online to GBMSM living with HIV. Participants in the intervention arm receive 10 video vignettes grounded in social learning and social cognitive theories that are designed to elicit critical thinking around issues of HIV transmission and disclosure. Participants in the attention control arm receive 10 video vignettes that focus on healthy living. All videos are optimized for mobile viewing. The study protocol includes five online assessments conducted over a 1-year period among 1500 US white, black, or Hispanic/Latino GBMSM living with HIV who report suboptimal antiretroviral therapy (ART) adherence or a detectable viral load in the past 12 months and recent CAS (past 6 months) with HIV-negative or unknown status male partners. Compared to the control arm, we hypothesize that men who watch the intervention videos will report at 12-month follow-up significantly fewer serodiscordant CAS partners, increased HIV disclosure, and improved social cognition (eg, condom use self-efficacy, perceived responsibility).

**Results:**

Participant recruitment began in June 2015 and ended in December 2015.

**Conclusions:**

This protocol describes the underlying theoretical framework and measures, study design, recruitment challenges, and antifraud measures for an online, video-based randomized controlled trial that has the potential to decrease HIV transmission risk behaviors among HIV-positive GBMSM who struggle with ART adherence. The Sex Positive!^[+]^ intervention allows for participation through multiple Internet-based mediums and has the potential to reach and engage a broader population of HIV-positive GBMSM who are virally unsuppressed.

**ClinicalTrial:**

ClinicalTrials.gov NCT02023580; https://clinicaltrials.gov/ct2/show/NCT02023580 (Archived by WebCite at http://www.webcitation.org/6iHzA8wRG)

## Introduction

Gay, bisexual, and other men who have sex with men (GBMSM) accounted for 67% of new human immunodeficiency virus (HIV) infections in the United States in 2012 [1]. Although antiretroviral therapy (ART) has improved survival with HIV, the low level of ART adherence presents a significant public health challenge in terms of the potential to transmit HIV [2]. Further, only three of the 96 evidence-based interventions defined by the Centers for Disease Control and Prevention have been designed for GBMSM living with HIV [3]. Among GBMSM with diagnosed HIV in the United States and Puerto Rico in 2010, it was estimated that 74% of men aged 18 to 24 years were virally unsuppressed compared to less than 40% in men aged 55 years and older. By race/ethnicity, the highest proportion of virally unsuppressed cases were among black GBMSM (63%), followed by Hispanic/Latino (58.5%), and white men (56%) [4]. This is particularly concerning because a recent study found that, compared to heterosexual males with HIV, GBMSM living with HIV who had unsuppressed viral loads had eight times the odds of engaging in serodiscordant condomless sex [5].

Preventing transmission in virally unsuppressed GBMSM who have condomless anal sex (CAS) with serodiscordant partners can have a great public health impact. Nationally, white, black, and Hispanic GBMSM continue to account for approximately 95% of newly diagnosed HIV infections among men [6]. Further, black and Hispanic men are also overrepresented as being virally unsuppressed [5,7,8]. Interventions designed to reduce CAS among GBMSM should be cost-effective and scalable, which are goals of the National HIV/AIDS Strategy [9]. Because new infections in GBMSM have been attributed in part to increased access to sex partners online [10,11], it is critical to deliver online behavioral interventions to GBMSM living with HIV to reach many high-risk men at a relatively low cost [12], engage men where they meet sex partners [13], and enable men to participate privately on a computer, tablet, or mobile phone with smartphone capabilities on their own schedule versus in a structured clinical setting [14].

### Users of Technology

Primarily because of the anonymity and accessibility of online spaces (websites, mobile apps), GBMSM have been early adopters of technologies designed for sexual partnering [15]. However, as promising as technology may be for HIV prevention and because Hispanic/Latino and black populations are more likely to own mobile phones and use mobile apps compared to white populations [16,17], HIV prevention technology also introduces often-overlooked methodological pitfalls such as low engagement of racial/ethnic minorities and recruitment bias. Historically, online HIV prevention work has had low representation of minority GBMSM [18-20]. There are two likely explanations for this disparity. First, black men constitute 13% of the US male population [21], which may account for the smaller proportion who complete online surveys. Second, online recruitment bias may occur, such that researchers may not be using targeted language or graphics, or recruiting from sites that cater to minority GBMSM [22].

### eHealth Interventions

Electronic health (eHealth) interventions have the potential to reach and engage GBMSM living with HIV from diverse racial/ethnic groups and socioeconomic statuses. These interventions are critical for risk-reduction efforts [23,24], can reach geographically dispersed men [12,25], and can adapt offline interventions [26]. However, few interventions have demonstrated efficacy in reducing HIV transmission from GBMSM who are HIV-positive. Recent eHealth and mobile health (mHealth) interventions for HIV-positive populations have focused on ART adherence [27,28], rather than sexual risk [29], and have been delivered through text messaging and on computers [30-33]. eHealth assessments of GBMSM living with HIV have been used to tailor provider-delivered interventions to decrease CAS with serodiscordant partners. Nevertheless, a recent provider-delivered eHealth intervention had low participation and retention, due mainly to factors such as time commitment and clinical setting [34]. Other eHealth interventions address HIV disclosure and condom use among GBMSM who are HIV-positive but are still in early stages of implementation (eg, Miranda et al [35]).

### Cost-Effectiveness of eHealth Interventions

There is a great need for cost-effective HIV prevention strategies for HIV-positive populations, particularly GBMSM who constitute the majority of those living with HIV [36,37]. Even modest intervention effects can have a significant public health impact because the two most important factors that determine cost-effectiveness are (1) the HIV prevalence of the target population (preventing transmission from men who are HIV-positive rather than preventing acquisition by men who are HIV-negative) and (2) the cost per person reached [38]. Effective HIV prevention interventions that use digital media are also likely to be highly cost-effective because they can be easily replicated after development, require minimal staffing, and have unlimited geographic reach [31,38-40].

### Aims and Objectives

The aim of this paper is to describe the study protocol of a randomized controlled trial (RCT) evaluating the effectiveness of a video-based messaging intervention delivered online, by comparing intervention and attention control groups on reductions in serodiscordant CAS and increases in HIV disclosure to sex partners. Through our prior work [18,19,41], we have identified a risk-reduction intervention approach for GBMSM living with HIV. The Sex Positive!^[+]^ study is conducted over a 1-year period among 1500 US white, black, and Hispanic/Latino GBMSM who were virally unsuppressed at some point during the past year, or who report suboptimal adherence to ART, and report recent CAS with serodiscordant male partners. Sex Positive!^[+]^ encompasses many characteristics found to reduce risk among HIV-positive populations in that it is theory-driven, has intervention content focused on HIV transmission behaviors, uses videos that demonstrate risk reduction and health behaviors through modeling, is delivered in an intensive manner, and is delivered over a 1-year period [42].

## Methods

### Trial Design

Sex Positive!^[+]^ is a two-arm randomized controlled phase III clinical trial with a 1:1 allocation ratio.

### Ethics Statement

The Institutional Review Board at Public Health Solutions in New York, NY, approved all study procedures. A waiver of documentation of written consent was obtained given the Internet-based research approach. Although Federal regulation requires that researchers obtain written informed consent for research on human subjects, under 45 CFR § 46.117(c) [43], written consent can be waived for research that involves minimal risk to participants and involves no procedures for which written consent is normally required outside of the research context. This research meets that criterion and we use an alternative approach where participants click a button signifying that they have read the informed consent page and agree to participate in the study. An advantage of online studies is that the consent form is available for the participant to review and/or print at any time. This strategy complies with the requirement of 45 CFR § 46.117(c) that participants are given a written statement describing the research and risks.

A Data and Safety Monitoring Board has been established to conduct semiannual reviews of study activities and to ensure participant safety, validity, and integrity of the data. The Board is comprised of experts, independent of the trial or funding agency, in RCTs, Internet research, Web design, and HIV-positive populations. Furthermore, a Certificate of Confidentiality has been obtained from the National Institute of Mental Health to help protect the privacy of HIV-positive participants enrolled in this health-related study.

### Participants

The target sample is 1500 high-risk, virally unsuppressed or less than 90% ART-adherent, US white, black, and Hispanic/Latino GBMSM living with HIV. Individuals participating in any aspect of the study must (1) be biologically male, (2) be age 18 or older, (3) be able to read and respond in English, (4) reside within the United States or a US territory, (5) report CAS with any HIV-negative or unknown status (serodiscordant) male partners in the past 6 months, (6) identify as HIV-positive, (7) report a detectable viral load, not being on ART and not knowing their viral load in the past year, or an undetectable viral load but less than 90% ART-adherent in the past 30 days, (8) identify as white, black, or Hispanic/Latino, (9) be willing to participate in an online intervention study for 12 months, and (10) have a working email address and mobile phone for intervention follow-up. We use quota sampling and targeted recruitment to ensure balanced representation of white (n=500), black (n=500), and Hispanic/Latino (n=500) men. Further, we include the following black racial/ethnic categories: black, African American, Caribbean, African, or multiethnic black [44]. In addition, we use targeted recruitment to ensure that 20% of the sample are men between the ages of 18 and 29 years. This group is overrepresented in the current HIV epidemic, particularly young men of color, and is less likely to be adherent to HIV medications or in care [4]. Men who meet study criteria, consent, and register to participate are automatically randomized into one of the two study arms.

### Recruitment

Men are identified for the study through social networking websites and gay-oriented sexual networking websites, dating websites, global positioning system (GPS)-based mobile phone apps that utilize targeted recruitment by city, race, and ethnicity, and online bulletin boards. By recruiting from different types of websites and mobile phone apps, we increase our chances of reaching a broader, more diverse pool of men with HIV. The goals of recruitment are to identify eligible participants online who are willing to participate in an online, longitudinal HIV risk-reduction intervention. Based on previous research findings [22], study banner advertisements mirror the racial/ethnic composition of each study subgroup. One of our sources of recruitment is POZ Personals, the dating site for *POZ Magazine*, which distributes internal system messages to a defined subset of US male members who are HIV-positive, at least 18 years of age, and self-identify as gay or bisexual.

### Primary Outcome Measures

Both HIV status disclosure to sex partners and serodiscordant CAS are assessed at each of five survey time points (baseline, 3-, 6-, 9-, and 12-month follow-up). The recall period for the primary outcome measures is the past 3 months for each of the five online assessments (Table 1).

**Table 1:** Survey instrument summary of primary outcomes assessed at baseline and at 3-, 6-, 9-, and 12-month follow-ups.

Primary outcomes measures	Description or lead question	Items
Serodiscordant condomless anal sex	Past 3 months, three most recent male sexual partners in one-on-one encounters	Sexual behavior by partner type (eg, one-time, repeat, exchange): insertive and/or receptive oral sex with or without condoms and ejaculation (y/n), insertive and/or receptive anal sex with or without condoms and ejaculation (y/n), drug and/or alcohol use prior to or during sex (y/n), total number of anal sex acts (with or without condoms) for each sex partner
HIV disclosure	Past 3 months, HIV disclosure with three most recent sex partners in one-on-one encounters	Demographic questions related to most recent partner(s) include: race/ethnicity, age, partner relationship type, partner serostatus, one-time vs repeat partner, and exchange vs nonexchange partner. HIV disclosure questions include: knowing partner(s) serostatus before or after having sex, asking partner(s) status, telling partner(s) one’s serostatus, who disclosed their serostatus first (participant or partner), how they learned about the partner(s) serostatus (eg, asking, telling, online profile)

### Secondary Outcome Measures

Secondary outcomes assessed at each time point include self-reported adherence to HIV medications, viral load, and CD4 count. Syndemic factors, or co-occurring epidemics, thought to be related to HIV transmission risk will also be assessed at various survey time points, including drug and alcohol use [45,46], depressive and anxiety symptoms [47-49], condom use and HIV disclosure self-efficacy [50], sexual compulsivity [51], HIV stigma, and interpersonal violence [52-54]. Process measures are used to track participants’ interest in, acceptability of, and verification of video viewing by having men complete brief postvideo online surveys. These surveys are designed to assess participants’ likes and dislikes as well as to elicit critical thinking about the video as it pertains to study outcomes [19,55].

### Sample Size

Based on the prevalence of behaviors in our previous studies, we calculated true proportions and sample sizes using chi-square tests for this two-arm design. We estimated that by enrolling approximately 750 men per group and retaining 75% at 12-month follow-up, we would have 80% power at a 5% alpha level to detect a minimum reduction of 8% in the number of serodiscordant CAS partners between the intervention and attention control arms.

### Intervention Content

Video messages are an effective way to deliver HIV prevention to GBMSM [19,56-58]. The first of our three theoretically grounded HIV prevention videos (from the HIV Big Deal project) tackling issues of CAS, HIV disclosure, and testing was rigorously evaluated among HIV-negative, HIV-positive, and untested GBMSM recruited online [19]. In our single-session video pilot for 971 GBMSM, we found significant reductions in CAS in the most recent encounter (9% decrease) and significant increases in HIV disclosure at 3-month follow-up (13% increase) compared to baseline [19]. In our subsequent online, single-session RCT for 3092 GBMSM that used videos from the HIV Big Deal project, we found significant reductions in CAS among men in the video study arm at 60-day follow-up (8% decrease) compared to baseline; HIV-positive men in the video study arm reduced their CAS (14% decrease), including with HIV-negative or status unknown partners, at 60-day follow-up (13% decrease) compared to baseline. Men living with HIV were also significantly more likely than men who were HIV-negative or untested to complete follow-up (57% vs 51%, *P*=.002) [18].

#### Theoretical Framework for the Intervention Videos

Employing a dramatic video series grounded in social learning and social cognitive theories [59,60], the Sex Positive!^[+]^ study engages learners through storytelling and promotes critical thinking on issues of HIV disclosure to sex partners, medication adherence and viral suppression, and serodiscordant CAS. In collaboration with a local production team, including a scriptwriter, producer, and director, we produced *Just a Guy*, a 6-episode video series that follows the story of “Guy,” an openly gay man living with HIV in Brooklyn, NY. The video series is based, in part, on the HIV Big Deal project described previously, which was launched online in 2008 [61]. According to social learning theory, individuals learn through the observation of others’ attitudes, behaviors, and the outcomes of those behaviors [59]. Videos developed for the intervention described in this paper include elements of social learning and attitude change theories, both of which informed the instructional design and delivery of our pilot online video intervention [19] and online feasibility trial of GBMSM [12]. More specifically, the intervention relies on three critical design dimensions including the medium, the degree of realism, and modeling (Table 2).

**Table 2:** Survey instrument summary of theoretical constructs assessed at baseline and at 3-, 6-, 9-, and 12-month follow-ups.

Construct, topic, and lead questions						Response options
**Construct: self-efficacy**						
	**Topic: disclosure to sex partners**					
		**Lead question: How confident are you that you could tell a potential sex partner your HIV status...**			Not at all confident, not very confident, somewhat confident, very confident, extremely confident	
			...in your online or mobile phone app dating profile?			
			...in an email?			
			...in a text message?			
			...over the phone?			
			...in person?			
	**Topic: safer sex [91]**					
		**Lead question: Now think about future sexual encounters with HIV-negative or unknown HIV status male partners. How confident are you that you could have anal sex with a condom...**			Not at all confident, not very confident, somewhat confident, very confident, extremely confident, prefer not to answer	
			When you feel depressed?			
			When you think that your partner does not want to use condoms?			
			When you are drunk or high on drugs?			
			When you are really sexually aroused?			
**Construct: self-regulatory skills**						
	**Topic: sexual compulsivity [92]**					
		**Lead question: Below are statements about sex that you may agree or disagree with.**			Not at all like me, slightly like me, mainly like me, very much like me, prefer not to answer	
			My sexual appetite has gotten in the way of my relationships.			
			My sexual thoughts and behaviors are causing problems in my life.			
			My desires to have sex have disrupted my daily life.			
			I sometimes fail to meet my commitments and responsibilities because of my sexual behaviors.			
			I sometimes get so horny I could lose control.			
			I find myself thinking about sex while at work.			
			I feel that sexual thoughts and feelings are stronger than I am.			
			I have to struggle to control my sexual thoughts and behavior.			
			I think about sex more than I would like to.			
			It has been difficult for me to find sex partners who desire having sex as much as I want to.			
**Construct: outcome expectancies**						
	**Topic: condoms and anal sex (adapted from Bimbi et al [93])**					
		**Lead question: Below is a list of statements that you may agree or disagree with.**			Strongly agree, somewhat agree, neutral, somewhat disagree, strongly disagree, prefer not to answer	
			I am more likely to use a condom with men who are HIV-negative or of unknown status.			
			I am more likely to have anal insertive sex (top) without a condom while drinking or high.			
			I am more likely to have anal receptive sex (bottom) without a condom while drinking or high.			
			I am less likely to have anal sex with men who are HIV-negative or of unknown status.			
**Construct: perceived responsibility**						
	**Topic: personal and partner responsibility for preventing HIV transmission [94]**					
		**Lead question: Below is a list of statements that you may agree or disagree with.**			Strongly agree, somewhat agree, neutral, somewhat disagree, strongly disagree, prefer not to answer	
			It is very important for me to use condoms to protect my sex partners from HIV.			
			HIV-positive gay men have a responsibility to keep other gay men from becoming positive.			
			When HIV-positive and HIV-negative men have sex with each other, they have an equal responsibility for being safe.			
			HIV-positive gay men have a special obligation to have safe sex with men who are negative or do not know their HIV status.			
			I feel responsible for protecting my partners from HIV.			
			If my partner is HIV-negative, he should not put the responsibility on me for safer sex.			
			If men who are HIV-negative want to have risky sex, it is their choice to do so.			
			It should be the responsibility of someone who is HIV-negative—not someone who is positive—to make sure their sex is safe.			
			I feel it is my partner’s responsibility to protect himself from HIV if he is negative.			
			It is my responsibility to protect others from getting HIV.			

Increasingly, eHealth HIV behavioral interventions are incorporating digital media, ranging from brief, untailored video interventions to complex computer-tailored multimedia interventions that target individual behaviors [62,63]. Online video-based interventions are an appealing and effective medium to deliver HIV prevention content to GBMSM [56,57]. Furthermore, video has greater potential to engage learners than conventional text or graphics in Web-based or print materials [64-66].

Storytelling, often more effective than exposition, is characterized by realism. From the perspectives of social learning and social cognitive theories [67], and attitude change theories [68], plausible “stories like mine” and “characters like me” are critical factors for engagement [69,70]. A Community Advisory Group, assembled for the Sex Positive!^[+]^ study, recommended that the videos feature an HIV-positive main character who overcomes a “victim” status and develops a sense of empowerment that positively impacts his personal relationships and physical health.

Social learning and cognitive theories emphasize the role of outcome expectancies regarding HIV disclosure and condom use self-efficacy and modeling of self-regulatory skills [59,60,71-73]. The tenets of social learning and social cognitive theories are embedded in the content, dialog, and nonverbal cues of the intervention videos, with the goal of preventing risk behavior before it happens. This intervention aims to reduce sexual risk behaviors by modeling HIV disclosure and discussions about safer sex. For example, in *Just a Guy*, the viewer learns through a nonverbal cue that Guy and Matt—a potential love interest—used a condom for anal sex the night before as Matt places the condom wrapper on his forehead on waking up. In this scene, Matt learns that Guy is HIV-positive and the two have a heated discussion about HIV disclosure responsibility. The video does not attempt to answer the question of personal responsibility or assert any one behavioral prescription, but rather encourages the viewer to think critically and discuss the issue with their sex partners.

#### Attention Control Videos

The two study arms are designed to be equal in the number of sessions, video length, study duration, and interest level. All videos are available for free viewing on the Sex Positive!^[+]^ study website, although men can view only videos that are assigned to their study arm. The control arm receives 10 healthy living videos that cover a range of topics including nutrition, physical exercise, smoking, and sleep quality. Attention control videos were selected from video-sharing websites and voted on by members of the research team.

#### Video Boosters

Because the effects of most preventive interventions tend to gradually wane over time, the inclusion of follow-up booster sessions can support prior skills learned to sustain an intervention impact [74]. Based on our team’s experience with intervention effects attenuating at 6 months [75], participants in the intervention arm will receive four video boosters after they complete the 6-month assessment survey. We edited *Ask Me, Tell Me* [76], the fourth video installment of our HIV Big Deal series, into three booster episodes (each episode is approximately 3 minutes in length). This particular prevention video from the HIV Big Deal series was selected for its emphasis on the importance of discussing one’s history of sexually transmitted infections (STIs) with sex partners and reducing episodes of CAS with serodiscordant partners. The final booster video focuses on the issue of social support for persons living with HIV and was selected from a video-sharing website. Participants in the attention control arm receive four additional healthy living videos after they complete the 6-month assessment survey.

### Intervention Activities

#### Overview

Participants in the intervention and attention control arms complete assessments at baseline and at 3-, 6-, 9-, and 12-month time points. To reduce the chance of instrument reactivity, we provide detailed online survey assessments at baseline and 12-month follow-up and brief assessments at 3-, 6-, and 9-month follow-ups. Participants will receive a text message and/or email with a survey link when it is time for them to complete a follow-up survey or watch a video. The dissemination of intervention and attention control videos occurs between the baseline and 3-month assessments, spaced 1 week apart. Following the 6-month assessment, participants in both arms receive four video boosters, spaced 1 week apart. The intervention and attention control videos are only available to study participants via a secure URL and men cannot forward video links to anyone, thus preventing potential cross-contamination between arms. All intervention activities occur online and are optimized for mobile performance.

#### Administrative Platform

For complex online interventions, developing a user-friendly administrative platform for the deployment and monitoring of data collection and intervention activities is critical. The online administrative platform enables study staff to screen potential participants, obtain consent, register, randomize participants into one of two study arms (intervention or attention control), monitor recruitment and retention, and flag suspicious cases. The administrative platform can be programmed to produce reports on participants, such as who completed certain study activities, who needs to be sent incentives, who needs to receive retention calls, and so forth. In addition, the study dashboard that participants see when they log in provides information on what study activities they have completed or need to complete, their personal information (eg, name, address, phone number) that can be updated, their communication preferences (eg, receiving texts or calls), and how much in Amazon.com incentives they have earned. The dashboard can also host hyperlinks to provide health information (eg, nutrition and HIV, ART adherence) and track which links participants click on.

#### Eligibility, Screening, and Consent

Men who click on a study banner, email, or online classified advertisement are directed to a brief, secure screener survey housed on the online administrative platform. Those who are determined to be eligible for study inclusion are directed to the study landing page and registration platform, which describes the study and provides a consent form for intervention activities. Men who are determined to be ineligible are provided with a message indicating that they are not eligible, thanking them for their time, and directing them to HIV prevention and other health resources, including an invitation to join a participant registry for future study opportunities.

#### Registration, Verification, and Randomization

After consent, participants are guided through online study registration, including the creation of a log-in username and password and collection and automated verification of their email address and mobile phone number. Then, they are automatically randomized into the intervention or attention control arm [77] through a stratified block randomization (by race/ethnicity and age), which will balance groups within a 5% range [77,78]. On accessing their study account, men are instructed to complete the baseline survey, which is hosted on a secure server on Survey Gizmo that is compliant with the Health Insurance Portability and Accountability Act. Men remain in their original assignment group (intention to treat) and are sent text message and email notifications for each of their intervention activities even if they discontinue participation.

#### Remuneration

**Figure 1:**
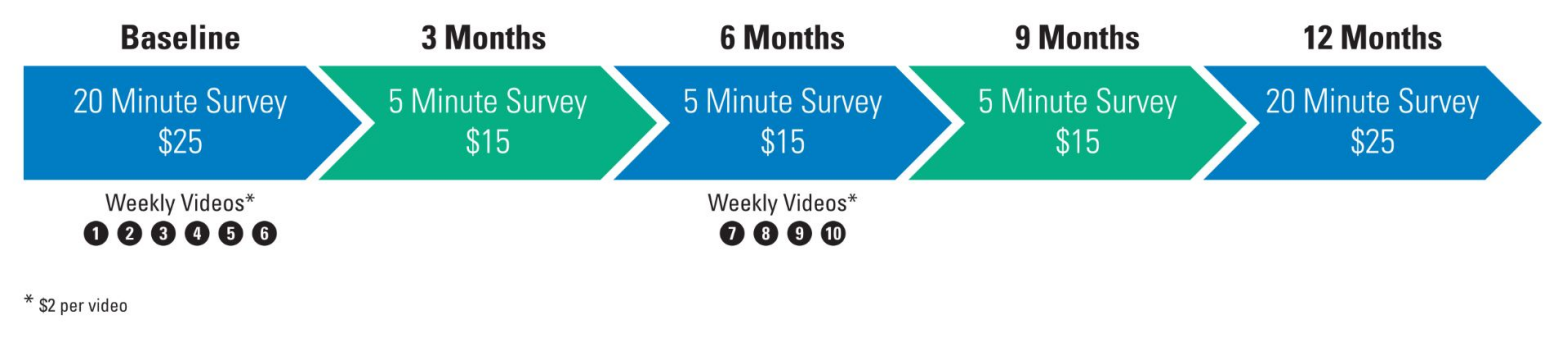
Study incentive structure.

Participants can receive up to a total of US $115 in Amazon.com gift cards (distributed electronically and via direct mail). Figure 1 provides the incentive schedule for Sex Positive!^[+]^.

#### Study Retention

Historically, online research has had lower retention rates than offline research because there are fewer social constraints compared to in-person interviewing [79]. However, recent advances in retention protocols and technology have greatly improved researchers’ ability to retain participants in HIV prevention trials, with 90% retention at 6 months and 82% retention at 12 months [80,81]. To ensure minimal attrition, Sex Positive!^[+]^ conducts multiple sessions, offers video boosters to maintain study interest, provides incentives for each study activity, sends reminder emails and text messages for videos and follow-up assessments, and uses the online administrative platform to create daily lists of nonresponsive participants for the retention coordinator to contact.

### Protection Against Fraud

An advantage of online research is data validity for sensitive information. Indeed, a growing number of studies indicate higher reporting of sexual risk and substance-using behaviors with computer-based surveys compared to mail, phone, and in-person surveys [82-84]. However, compared to the gold standard of in-person interviewing, a limitation of online research—as with mail and phone surveys—is the challenge of verifying a participant’s identity [85]. Based on recommendations made during an open session of our Data and Safety Monitoring Board, as well as a meeting of Internet experts about the issue of online fraud in research studies [86], the Sex Positive!^[+]^ study implements several protections aimed at reducing the likelihood of fraud. Specifically, (1) contact information is verified during registration using multiple methods; (2) duplicate detection (of Internet Protocol [IP] address and mailing address) software is used to detect instances of participants attempting to create multiple study profiles; (3) mobile phone numbers are investigated to determine whether they are voice over IP (VoIP) numbers—an individual can obtain multiple VoIP numbers on the Internet, typically for no charge, that can be routed to the individual’s mobile device (eg, this helps a potential participant to sign up numerous times with unique phone numbers); (4) proxy IP addresses and invalid mailing addresses are flagged for further scrutiny by the research team; (5) trap questions are used in the baseline survey to flag cases with inconsistent data—“I am HIV-negative”—or careless responses (eg, straightlining); (6) study staff conduct weekly analyses of new screener data to identify individuals (by IP address) who make multiple attempts to join the study; (7) compensation is kept sufficiently low to reduce the chances of participating solely to gain incentive payments; and (8) to ensure participant authenticity, the final study incentive is mailed to a verified physical address.

### Analysis

Primary analyses for intervention efficacy will examine whether participants in the intervention arm report fewer CAS acts with serodiscordant partners, a higher percentage of anal sex acts with condoms, fewer sexual partners, and more disclosure of HIV status with partners compared to participants in the attention control arm. Dose-response analyses will allow us to examine whether a certain number of intervention videos—“doses”—are necessary to effect study goals.

Even with a robust retention plan, incentives, and survey programming that requires responses, missing data are inevitable because participants can “refuse” survey items or drop out of the study. For this study, data from the online screener will verify that blocked randomization produced equivalent groups and will also be used to assess possible sample attrition bias. Although there are many ways to handle missing data, our experience suggests that maximum likelihood estimation is the best approach, using the appropriate algorithm for estimation purposes.

We will also conduct analyses to assess the savings in averted HIV-related lifetime treatment costs, the total number of quality-adjusted life years (QALYs) saved by preventing a single HIV infection, and the cost of developing and implementing the Sex Positive!^[+]^study. In general terms, after the 12-month follow-up we will analyze up to three partner-by-partner sexual encounters, for biological male partners only, as well as global number of condomless anal or vaginal sex partners (ie, biological male and female partners, transidentified females, and transidentified males) at each of the four follow-up assessment time points. This will provide an estimate of the number of secondary infections expected among the participant’s serodiscordant sex partners. The main analysis will assume that study participants with an undetectable viral load at a particular time point are noninfectious. Although a separate estimate will be calculated for each participant at each of the four follow-up time points, the sum of these time point findings will estimate the total number of secondary infections expected among the participant’s sex partners during the entire study period. We will then calculate the total number of estimated infections prevented by the intervention arm versus the control by measuring the difference in the mean number of expected secondary infections for all men in the intervention and control arms over the 12-month period. Based on the total number of prevented infections, we will calculate the corresponding savings in averted HIV-related lifetime treatment costs as well as the total number of QALYs saved by preventing a single HIV infection. Finally, we will calculate the net cost of the intervention, the cost per infection prevented, and the net cost per QALY saved (ie, the cost-utility ratio) [87]. The intervention can be considered “cost saving” if the net cost is negative and “cost-effective” if the cost-utility ratio is less than US $100,000 per QALY saved [88].

## Results

Participant recruitment began in June 2015 and ended in December 2015.

## Discussion

Those eHealth interventions, such as Sex Positive!^[+]^, that allow participation through multiple Internet-based mediums (ie, computer and mobile access) have the potential to reach and engage a broader population of GBMSM with HIV. More specifically, this type of online intervention can reach men living with HIV who are outside of HIV epicenters, who may be beyond the reach of traditional prevention services, and are poorly represented in research. Furthermore, the online administrative platform and videos will be accessible to a much larger population at a relatively low cost following completion and evaluation of the study. For populations with limited Internet access, the intervention can be adapted for use in HIV clinics and community-based organizations via private kiosks, laptops or tablets, or in small group settings. Thus, this self-administered, online video-based intervention can be implemented in various settings at minimal cost.

### Limitations

This study protocol has several limitations that deserve mention. All men were recruited online, through social networking and gay-oriented sexual networking websites and mobile phone apps. As such, the findings may not be generalizable to HIV-positive GBMSM who do not own a mobile phone or have Internet access, access these types of websites or mobile phone apps, to men who do not identify as gay, to individuals exposed to a study banner or email but choose not to click on it, or to men who do not identify as black, white, or Hispanic/Latino. Study content is only available in English, which limits its reach to participants. There is a need to translate content into Spanish because it is the second most-spoken language in the United States and represents a subpopulation of GBMSM with high rates of HIV [4,89]. Lastly, a potential limitation is that participants self-report their health outcomes, specifically viral load. However, a recent validation study of 639 individuals with HIV from an ongoing prospective study in New York found that participant recall of viral load agreed with the Department of Health’s registry data 85% of the time [90].

### Conclusion

In conclusion, the Sex Positive!^[+]^ study addresses the lack of interventions designed for GBMSM living with HIV. This protocol describes the underlying theoretical framework and measures, study design, recruitment challenges, and antifraud measures. If efficacious, it will have a significant impact on reducing HIV transmission risk in a disproportionately affected population. Although this eHealth intervention is being implemented with virally unsuppressed men or men who struggle with ART adherence, it can be adapted for delivery in other settings and with other populations.

## Abbreviations

ART: antiretroviral therapy
CAS: condomless anal sex
GBMSM: gay, bisexual, and other men who have sex with men
GPS: Global Positioning System
HIV: human immunodeficiency virus
IP: Internet Protocol
QALY: quality-adjusted life years
RCT: randomized controlled trial
STI: sexually transmitted infection
VoIP: voice over Internet Protocol
